# Autistic Traits and Social Anxiety in Chinese College Students: The Longitudinal Mediating Role of Rumination

**DOI:** 10.1155/da/6103362

**Published:** 2025-10-01

**Authors:** Lulu Hou, Wendian Shi

**Affiliations:** ^1^School of Psychology, Shanghai Normal University, Shanghai 200234, China; ^2^Lab for Educational Big Data and Policymaking (Ministry of Education), Shanghai Normal University, Shanghai 200234, China

**Keywords:** autistic traits, latent growth model, rumination, social anxiety, structural equation model

## Abstract

**Background:**

Autistic traits (ATs) and social anxiety (SA) are closely associated; however, few studies have investigated the potential mediating mechanism of this relationship using longitudinal data. This study examined: (1) the developmental trajectories of ATs, rumination, and SA among college students; (2) whether the baseline levels of ATs predicted the developmental trajectories of SA; and (3) whether the trajectories of rumination mediated this longitudinal association.

**Methods:**

This study enrolled 397 college students to complete Broad Autism Phenotype Questionnaire, Discriminative Response Scale, and SA Disorder Dimension three times over the course of a year. Three unconditional latent growth models (LGMs) were first used to explore the trajectories of ATs, rumination, and SA, respectively. Then, a conditional LGM was used to examine the direct longitudinal association between ATs and SA. Finally, a structural equation model was further used to examine the longitudinal mediating role of rumination between ATs and SA.

**Results:**

For college students, ATs remained relatively stable, whereas rumination and SA declined across the study period. Furthermore, ATs positively predicted the intercept of SA (*β* = 0.66, *p* < 0.001), and negatively predicted the slope of SA (*β* = −0.29, *p* < 0.001). More importantly, higher baseline levels of rumination mediated ATs on the baseline value of SA (0.14, 95% confidence interval [CI] [0.08 0.22]), and slower rates of decline of rumination mediated ATs on the change in SA (−0.15, 95% CI [−0.46 −0.01]).

**Conclusions:**

These results indicate that college is a critical period for the abatement of rumination and SA. Furthermore, rumination might be one of the mechanisms underlying the link between ATs and SA. Interventions to prevent the negative impact of ATs might help to decrease the risk of rumination and SA in the college students.

## 1. Introduction

Social anxiety (SA) is characterized by the feelings of anxiety that emerge in response to the perception of being evaluated by others, either in real or imagined social contexts [1]. Using a cross-sectional design, Jefferies and Ungar [2] found higher SA scores for 18–24-year-olds groups than those both in 16–17-year-olds and 25–29-year-olds groups in a study targeting individuals aged 16–29 years. Among those in early adulthood, SA can be particularly challenging for college students [3, 4]. This may be because college campuses are highly social environments where individuals aspire to fit into that society, which causes some college students to experience severe anxiety and fear in social situations that may generate negative evaluations [5]. Previous studies have shown that the rate of severe SA in the college student population ranges from approximately 10%–30% [2, 6, 7] and that the level of SA among college students is increasing over time [8]. SA is not only associated with poor performance and lower quality of life in social situations [9–11], but also closely related to depression [12, 13] and substance abuse [14–16], whether for individuals diagnosed with SA disorder (SAD) or subclinical populations.

### 1.1. Development of SA in College Students

In order to prevent and intervene in SA more effectively, researchers have studied SA from a developmental perspective. As an example, McLaughlin and King [17] assessed a diverse sample of early adolescents aged 10–15 years in grades 6–8 at three time points across 1-year and found SA symptoms declined across the study period. Zimmer-Gembeck et al. [18] recruited adolescents aged 10–13 years and measured them seven times over the following 5 years until they were 15–18 years old. The study revealed that adolescents exhibited a significant quadratic pattern of growth in SA levels, with symptoms markedly increasing around the midpoint of the study, specifically when the participants were, on average, 14 years old. Recently, Jiao et al. [19] used a 12-week interval with three time points and found that SA remained stable in a short frame among U.S. college students. Although current research has not found the development of SA to be age-specific, the distinct social environment of college (e.g., nonfixed schedules, diverse elective courses, rich extracurricular/practical activities) differs significantly from adolescence [20], leading individuals to face more complex daily social challenges. Consequently, long-term longitudinal design is needed to thoroughly investigate the development of SA among college students. Specifically, the developmental characteristics of SA can be demonstrated through both its initial level and developmental rate. The former reflects the starting point of development, while the latter captures the trend and rate of the development process. Therefore, when investigating the development of SA, it is essential to consider both the initial level and the rate of development concurrently, as this approach can yield new insights for existing research.

### 1.2. Autistic Traits (ATs) and SA

Spence and Rapee [21] proposed that individual (e.g., behavioral inhibition and avoidance) and environmental factors (e.g., parental influences) interact to shape proximal behavioral (e.g., social skills) and cognitive (e.g., beliefs) factors that determine an individual's SA level. Among them, the influence of personality on SA has received extensive attention from researchers [22, 23]. For example, studies found neuroticism and extraversion could be used to predict SA [24, 25]. In addition, as a series of personality traits different from the big five personality, ATs have also received attention from researchers [26, 27]. ATs represent a dimension of individual differences characterized by variations in social communication, cognitive styles, sensory processing, and behavioral patterns commonly associated with autism spectrum disorder (ASD), but existing on a continuum across the entire population, irrespective of clinical diagnosis [28–30]. ATs encompass features such as differences in social cognition, a preference for predictability, concentrated interests, and atypical sensory sensitivities [31–34]. While ASD diagnosis [35] requires clinically significant impairment from such traits, ATs themselves represent subclinical variations that can be observed in both clinical and nonclinical populations and are not inherently pathological. Previous studies have shown that ATs are linked to SA among college student populations [36, 37].

The close relationship between ATs and SA may be related to the differences in social communications and empathy characteristics of individuals with high ATs. Specifically, the differences in social communications made those with high ATs more susceptible to rejection from peers [38] because maintaining harmony through consistency with others is very important in Chinese culture. Furthermore, Zhao et al. [39] found that individuals with high ATs exhibited lower levels of cognitive empathy and empathy concerns but higher levels of personal distress (also known as affective sharing or empathic accuracy). On the one hand, reduced cognitive empathy hinders their ability to perceive others' intentions and views on themselves in social activities. On the other hand, the increased personal distress causes them to feel negative when facing the disadvantaged situation of others, leading to their avoidance and anxiety towards social activities, which has been confirmed in previous empirical studies [40, 41]. Taken together, two aspects in empathy contribute to the heightened level of SA in individuals with high ATs.

Despite previous studies demonstrating a strong relationship between ATs and SA, several gaps remain in the existing literature. First, previous studies do not address the trajectory of ATs. As previously noted, both the initial level and the rate of development offer valuable insights into the status of ATs among college students. This information enhances our understanding of the changes in ATs during the university experience. Second, studies focused on the relationship between ATs and SA used cross-sectional designs [36, 37], which fail to address the parallel processes between ATs and SA, as well as their long-term trajectories. In other words, previous studies have only shown a correlational relationship between ATs and SA levels at the same time point. However, it remains unclear whether a high initial level of ATs can accelerate the increase in SA levels or slow the decline of SA levels, thereby acting as a catalyst for changes in SA. Clarifying this relationship will enhance our understanding of the role of ATs in the development of SA. Similarly, it is also important to investigate whether the developmental rate of ATs influences the development of SA. Finally, it is also unclear whether the longitudinal predictive effect of ATs on SA is mediated by additional factors. Investigating potential mediating mechanisms may facilitate intervention in these variables, thereby weakening or severing the strong association between ATs and SA. This intervention would help mitigate the adverse development of SA in individuals displaying elevated levels of ATs.

### 1.3. Rumination as a Potential Mediator

White et al. [42] proposed a comprehensive developmental model to discuss the frequent comorbidity of ASD and anxiety disorders, highlighting that impairments in emotion regulation serve as a fundamental mechanism linking them. This suggests that the heightened use of maladaptive emotion regulation strategies may serve as a significant link between ATs and SA within the general population.

As a well-researched maladaptive emotion regulation strategy, rumination is characterized by repetitive and passive focus on the symptoms of distress, as well as their potential causes and consequences [43]. On the one hand, previous research has shown that ATs are positively correlated with rumination [44]. Individuals with high ATs often experience personal distress in social activities, so their concerns about the struggles of others may be intertwined with their own fear of adverse outcomes [41]. Moreover, personal distress is a negative self-focused trait, leading individuals with high ATs to frequently engage in ruminative coping strategies. This tendency manifests not only in response to the suffering of others but also during solitary moments or in situations devoid of social interaction [45, 46]. On the other hand, previous cross-sectional studies have found that rumination is closely related to SA [47–49] and one study that used a longitudinal design found that baseline rumination scores predicted the level of SA in the following week [50]. Those individuals who repeatedly recalled negative events related to themselves formed negative expectations about subsequent social situations and experienced more anxiety. However, no studies have directly examined whether rumination also mediates the longitudinal effects of ATs on SA.

In addition, when exploring the longitudinal mediating role of rumination, it is necessary to examine the specific mechanisms by which the initial level and the developmental rate of rumination itself play a role in the trajectory between ATs and SA. This is because previous studies on the development of rumination have adopted the cross-sectional design and have not included college students. For example, Hampel and Petermann [51] investigated the coping styles of 1123 participants (8–14 years old) in late childhood (grades 3–4), early adolescence (grades 5–6), and middle adolescence (grade 7) groups. The results showed that the participants in grades 5–7 scored lowest in adaptive coping strategies and highest in maladaptive coping strategies (including rumination). However, it is still unknown how rumination changes in early adulthood and whether it continues to decline. Sütterlin et al. (2016) [52] recruited 299 participants ranging from 15 to 87 and found that those aged 63 years and older exhibited lower levels of ruminative thinking than other age groups. However, a small sample size and a large age range are not conducive to inferring the changing trends of rumination in early adulthood. This study considers a sample of college students, and thus provides an important complement to the two cross-sectional studies. Previous studies on the mediating effect of rumination were largely cross-sectional in design [44], which restricted the exploration of the mediating mechanism of rumination. To consider this question in detail, we must clarify the initial level and the developmental rate of rumination itself, and specifically answer which aspect plays the mediation role, or whether both do.

### 1.4. The Present Study

In summary, although previous studies have preliminarily explored the differences of rumination and SA across different age groups (e.g., [2, 52]) and investigated the trajectory of SA in adolescents [18] or within a short timeframe among college students [19], there has been no research examining the trajectories of ATs, rumination, and SA in the college student population through long-term longitudinal design. Second, although previous studies have revealed the association between ATs and SA [36, 37] in the general population, longitudinal designs have not been used to examine the relationship between their trajectories. Finally, although previous studies have found the close association between rumination and ATs [44] and between rumination and SA (e.g., [48]), no research has examined the longitudinal mediating role of rumination between ATs and SA. This study aimed to address these gaps by testing the mediation effects of trajectories of rumination in the relationship between trajectories of ATs and SA among college students. Due to the high stability of ATs [53], we hypothesized that ATs would remain stable, while rumination and SA would decline during early adulthood. In addition, we hypothesized that higher ATs would be associated with elevated rumination and SA at baseline and predict a slower rate of decline in their trajectories over time. Finally, we hypothesized that the associations of ATs with the trajectory of SA would be mediated by elevated rumination levels at baseline and a slower rate of decline of rumination.

## 2. Methods

### 2.1. Participants

Data were collected from a comprehensive longitudinal study examining the mental health of young adults in China. This study recruited college students aged 18–26 years through online advertisements and administered online using the Wenjuanxing platform. Participants were asked to complete anonymous self-report questionnaires on ATs, rumination, and SA three times between November 2020 and November 2021, with a 6-month interval between each survey (T1: November 15–30, 2020; T2: May 15–31, 2021; T3: November 15–30, 2021). Furthermore, all questions have been made mandatory. While participants have the option to withdraw if they find it challenging to answer certain questions, the valid questionnaires collected consist of complete data, with no missing values. Due to the limited number of literatures on the same model as this study and the lack of methods for calculating sample size, we followed the recommendation of the multiple mediation model with two parallel mediators (i.e., initial level and developmental rate in this study) and determined 250 people as the minimum sample [54]. Given the high attrition rate associated with longitudinal research, we gathered as much data as possible until November 30, 2020. Ultimately, a total of 592 individuals who expressed a willingness to participate were selected as the sample for T1. Then, 152 participants were lost by T2, and an additional 43 participants were lost by T3, resulting in a total loss rate of 32.94%. Consequently, the final analysis comprised 397 participants, of whom 272 were female, with a mean age of 21.43 years (standard deviation = 2.22; age range = 17.25–27.58 years at T1). The final sample included 48 freshmen, 100 sophomores, 68 juniors, 39 seniors (of whom three were fifth-year seniors), 135 graduate students, and seven individuals who had graduated and were preparing for graduate entrance examinations at home. Using the online program developed by Schoemann et al. [55], the post hoc statistical powers were greater than 0.90 in this study.

It should be noted that during the recruitment process, we required participants to be college students aged 18–26 years. However, in the final sample, there were 16 individuals who were under 18 or over 26 years old and seven individuals who were in the gap year before graduate school. Considering that the general education policy requires attending university at the age of 18 or 19 years, but the degree of implementation of education policies varies in different regions, there may be some age deviations that are acceptable. In addition, since these seven individuals in gap years did not attend work at all, we believe that they are also within the broad category of college students and were not excluded in the final analysis. We also provided the results of excluding these seven participants in Section 1 of Supporting Information S1.

This study received approval from the Ethics Committee of Shanghai Normal University. Before participating in the first test, all participants signed an informed consent form. All procedures involving human participants adhered to the ethical standards set forth by the institutional and/or national research committee, as well as the principles established in the 1964 Helsinki Declaration and its subsequent amendments or comparable ethical guidelines.

### 2.2. Measures

#### 2.2.1. Broad Autism Phenotype Questionnaire (BAPQ)

The BAPQ [56] was employed to measure ATs. This questionnaire is comprised of three dimensions (i.e., aloof personality, rigid personality, and pragmatic language), each containing 12 items, resulting in a total of 36 items. Participants rated their experiences on a scale from 1 (very rarely) to 6 (very often). Hurley et al. [56] demonstrated high internal consistency and reliability of the English version BAPQ. We employed the “Translation–Backtranslation–Revision” method to create a Chinese version one. Specifically, the first author of the manuscript, whose native language is Chinese but has published a large number of literatures in English and holds relevant qualifications in English language studies, translated the questionnaire from English to Chinese. Subsequently, another colleague, a native Chinese speaker with 5 years of experience in English-speaking countries and a doctoral degree in psychology from a European institution, conducted a back-translation of the questionnaire from Chinese to English. Following discussions, the final Chinese version of the questionnaire was revised. We conducted a confirmatory factor analysis on the questionnaire, and the results indicated that the factor loadings for several items (aloof personality: item 5; rigid personality: items 8, 13, 24, 26, and 35; pragmatic language: items 21 and 34) were below 0.30 at least at one time point. After excluding these items, there were 11, 7, and 10 items for the aloof personality, rigid personality, and pragmatic language dimensions, respectively. Moderate-to-high Cronbach's *α* coefficients (aloof personality: 0.87–0.90; rigid personality: 0.72–0.78; pragmatic language: 0.76–0.79; full scale: 0.88–0.89 across three time points) indicated good reliability of this scale in this study. Furthermore, the questionnaire demonstrated acceptable structural validity, with *χ*^2^/*df* ranging from 1.97 to 2.15, comparative fit index (CFI) from 0.895 to 0.908, Tucker–Lewis index (TLI) from 0.875 to 0.889, and root mean square error of approximation (RMSEA) from 0.050 to 0.054 across time points in this study.

#### 2.2.2. Social Anxiety Disorder Dimension (SAD-D)

The SAD-D [57] was utilized to assess SA. Participants rated 10 items from 0 (never) to 4 (all of the time). The SAD-D was developed in accordance with the core template established by the Anxiety Disorder Working Group of the fifth edition of the Diagnostic and Statistical Manual of Mental Disorders to assess anxiety [57]. This template employs a dimensional approach to measure anxiety symptoms, incorporating three response components associated with anxiety: verbal and cognitive reports of subjective fear, physiological responses, and avoidance behaviors. When assessing SA, this scale requires participants to report the frequency of these symptoms occurring in social situations. LeBeau et al. [58] have demonstrated that SAD-D possesses strong internal consistency, convergent validity, and discriminant validity in clinical samples. We translated it following the same procedure used for the BAPQ. We also conducted confirmatory factor analysis on SAD-D and found that the factor loadings of all items were above 0.30, so all 10 items were ultimately retained. In this study, Cronbach's *α* coefficients ranging from 0.90 to 0.91 across three time points. Furthermore, the questionnaire had a good structural validity, with *χ*^2^/*df* ranging from 1.16 to 1.53, CFI ranging from 0.990 to 0.997, TLI ranging from 0.983 to 0.995, and RMSEA ranging from 0.021 to 0.026 across time points in this study. Using the Interaction Anxiousness Scale (IAS; [59, 60]) as validity criteria, there were significant correlations between SAD-D and IAS scores across time points, with Pearson correlation coefficients ranging from 0.67 to 0.73.

#### 2.2.3. Ruminative Response Scale (RRS)

The RRS [61] was used to measure rumination. Participants rated 22 items from 1 (almost never) to 4 (almost always). The RRS consists of three dimensions: depression (12 items), reflection (five items), and brooding (five items). The Chinese version of the questionnaire has been proven to have good reliability in previous studies [62]. After confirmatory factor analysis, among the measurements at three time points, the factor load of item 12 on the brooding dimension was low, so it was possible to eliminate it. After the removal of the item, there were 12, five, and four items for the depression, reflection, and brooding dimensions, respectively. Cronbach's *α* coefficients ranging from 0.88 to 0.91 for the depression dimension, 0.74–0.80 for the reflection dimension, 0.75–0.80 for the brooding dimension, and 0.91–0.93 for the full scale across the three time points in this study. Furthermore, the questionnaire had a good structural validity across time points in this study, with *χ*^2^/*df* ranging from 1.75 to 2.34, CFI ranging from 0.930 to 0.942, TLI ranging from 0.911 to 0.932, and RMSEA ranging from 0.043 to 0.058.

#### 2.2.4. Data Analysis

We first used SPSS 22.0 to examine descriptive statistics and zero-order correlations for each of the three scales across all time points. Then, Mplus 8.0 was used to model the relationship among the variables in a series of four steps. In the first step, measurement invariance tests were conducted for each scale to determine whether they exhibited measurement invariance across different time points. The four sequentially nested models included configural invariance (baseline model), weak invariance (constraining factor loadings across time points), strong invariance (constraining intercepts across time points), and strict invariance (constraining residuals across time points). Because these models are sequentially nested, the next test can be performed only if the previous model's requirements are met. The ΔCFI is unaffected by sample size, which makes it the best indicator of model differences [63]. When |ΔCFI| ≤ 0.01, |ΔTLI| ≤ 0.01, and |ΔRMSEA| < 0.015, the difference between the two models may be disregarded [64]. In the second step, three unconditional linear latent growth models (LGMs) were fitted to examine the trajectories of the three variables (i.e., ATs, rumination, and SA) with two latent variables—an intercept factor describing the initial level and a slope factor describing the developmental rate. The factor loadings of the intercept were all fixed at 1, and the factor loadings of the slope were set to 0, 1, and 2 for each of the three time points, respectively, given the intervals between the time points were all equal. In the third step, a parallel LGM was used to examine the relationship between the trajectories of ATs and SA. It should be noted that since we assumed that ATs would remain stable, if the slope of ATs was not significant in the second step, then the conditional LGM rather than the parallel LGM would be used to model the effect of the ATs levels at baseline on the trajectory of SA. In the final step, a structural equation model (SEM) was employed to test the direct paths between the intercepts and slopes of the three variables. Moreover, a bootstrap procedure was implemented to evaluate the indirect effect and to obtain confidence intervals (CIs). Utilizing random sampling, we generated 1000 bootstrap samples from the original dataset. The CFI and TLI, along with the RMSEA were used to evaluate model fit. Generally, CFI and TLI need to be larger than 0.90 (0.85 is acceptable), while RMSEA should be less than 0.05 (0.08 is acceptable) [65–68]. Additionally, the maximum likelihood robust (MLR) estimator was utilized in all analyses to address potential issues arising from nonnormal data distributions.

## 3. Results

### 3.1. Preliminary Analyses

Table 1 presents the means, standard deviations, and intercorrelations for all the study variables. There were positive correlations among ATs, rumination, and SA symptoms at each time point (*p*s < 0.001).

### 3.2. Measurement Invariance Test

Pairwise comparisons of the four models of each scale exhibited support for metric invariance (|ΔCFI|s = 0.001, |ΔTLI|s ≤ 0.002, |ΔRMSEA|s = 0.001), scalar invariance (|ΔCFI|s ≤ 0.005, |ΔTLI|s ≤ 0.004, |ΔRMSEA|s ≤ 0.002), and strict invariance (|ΔCFI|s = 0.002, |ΔTLI|s ≤ 0.001, |ΔRMSEA|s ≤ 0.001), which suggested that the factor structure of all scales had good measurement invariance across different time points (Table 2).

### 3.3. Trajectories of ATs, Rumination, and SA

Results from the LGMs for the trajectories of ATs, rumination, and SA are presented in Table 3. These results showed that the fit indices for the three models were good, with the exception of the RMSEA. The slope for the ATs was not significant, indicating that no significant change in ATs occurred among college students between the three measurements. The slopes of rumination and SA were negative, indicating a decrease in rumination and SA during the study.

### 3.4. The Direct Effects of ATs on the Trajectory of SA

Due to the nonsignificant slope of ATs in the unconditional LGM, we subsequently used the conditional LGM rather than parallel LGM to model the effect of the baseline ATs levels on the trajectory of SA.

The conditional LGM with ATs at T1 as the independent variable, and the trajectory of SA as the dependent variable was found to fit well (*χ*^2^/*df* = 3.36, RMSEA = 0.077, CFI = 0.985, TLI = 0.970). ATs positively predicted the intercept of SA (*β* = 0.66, *t* = 23.87, *p* < 0.001), and negatively predicted the slope of SA (*β* = −0.29, *t* = −6.18, *p* < 0.001).

### 3.5. The Longitudinal Mediating Role of Rumination Between ATs and SA

**Figure 1:**
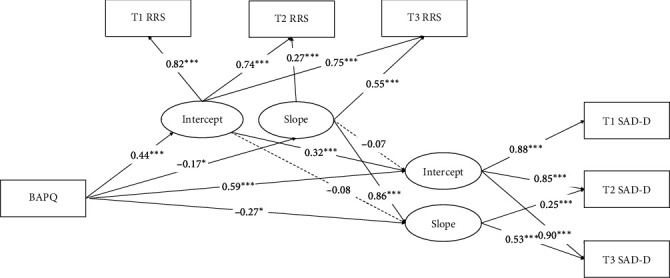
The longitudinal mediating role of rumination between autistic traits and social anxiety symptoms. BAPQ, Broad Autism Phenotype Questionnaire; RRS, Ruminative Response Scale; SAD-D, Social Anxiety Disorder Dimension. Solid lines indicate significant path coefficients, dashed lines indicate insignificant path coefficients; *⁣*^*⁣*^*∗*^^*p* < 0.05, *⁣*^*⁣*^*∗∗∗*^^*p* < 0.001.

The results of the SEM (see Figure 1) showed good fit indices (*χ*^2^/*df* = 3.83, CFI = 0.973, TLI = 0.943, RMSEA = 0.084). ATs positively predicted the intercept (*β* = 0.59, *t* = 11.58, *p* < 0.001), and negatively predicted the slope (*β* = −0.27, *t* = −2.01, *p*=0.045) of SA. Meanwhile, ATs positively predicted the intercept (*β* = 0.44, *t* = 7.29, *p* < 0.001), and negatively predicted the slope (*β* = −0.17, *t* = −2.12, *p*=0.03) of rumination. The intercept of rumination positively predicted the intercept (*β* = 0.32, *t* = 4.97, *p* < 0.001), but did not significantly predict the slope (*β* = −0.08, *t* = −0.64, *p*=0.52) of SA. Finally, the slope of rumination positively predicted the slope (*β* = 0.86, *t* = 3.59, *p* < 0.001), but not the intercept (*β* = −0.07, *t* = −0.93, *p*=0.36) of SA.

Furthermore, the Bootstrap results showed that the mediating effect of the indirect pathways of ATs → rumination intercept → SA intercept was 0.14, 95% CI [0.08 0.22], and that the mediating effect of the indirect pathways of ATs → rumination slope → SA slope was −0.15, 95% CI [−0.46 −0.01]. Because the 95% CI did not include 0, the two indirect pathways were both significant.

## 4. Discussion

In this study, a longitudinal analysis of the data from three different time points revealed that ATs remained stable in the college student population, while rumination and SA decreased. Our results indicate that higher levels of ATs were associated with elevated rumination and SA at baseline and with their slower decrease over time. The mediation analysis indicated that higher baseline rumination levels mediated ATs at baseline on the baseline value of SA, and a slower rate of decline of rumination mediated ATs at baseline on the change in SA.

### 4.1. Trajectories of ATs, Rumination, and SA

The result of the nonsignificant slope for the ATs over the 1-year period for college students was consistent with previous studies using samples of children, adolescents, and adult populations [69–72]. The high stability of ATs may be caused by the high heritability [71].

Furthermore, the negative slope of SA may come from several influencing factors. On the one hand, in order to prevent the spread of COVID-19, most universities adopted online teaching and learning in the spring semester of 2020 and asked students to stay at home as much as possible. Therefore, students' SA peaked in the autumn semester after returning to school [73, 74] due to the decreased social activities in the previous semester. So, a downward trend over time was observed in the following year. On the other hand, combing the results of Zimmer-Gembeck et al. [18], the trend of SA peaking and then subsequently declining in adolescence may be linked to decreased emotional responsiveness, enhanced emotional control, and psychological resilience resulting from changes in life circumstances, significant life events, and neurodevelopmental processes [75], [76]. It should be noted that the mean scores of SAD-D in this study were lower than the mean scores of 25.70 reported in American clinical samples [58] and the cutoffs of 18.50 established in an Australian sample by Rice et al. [77]. Consequently, while the observed decrease in SA over time is statistically significant, it may not be clinically relevant. In addition, the negative slope of rumination complemented the two existing cross-sectional studies [51, 52] and may benefit from the improved emotion regulation abilities as college students age [78]. The enhanced mastery and application of adaptive coping strategies, particularly cognitive reappraisal, can substitute for and diminish the frequency of rumination.

### 4.2. The Direct Effects of ATs on the Trajectory of SA

The results of the conditional LGM indicated that ATs predicted both the initial level and the rate of decline of SA. The results of the present study found that ATs were positively correlated with initial levels of SA, which is in line with two previous cross-sectional studies, which found a positive relationship between ATs and SA levels among college students [36, 37]. More importantly, by using a longitudinal design, the present study found that ATs significantly predicted the rate of decline of SA, which is an important addition to previous studies.

It should be noted that the findings of this study are seemly different from the results of Day et al. [79] for autistic youth. The study investigated SA in both autistic and nonautistic youth from June to December 2020, across seven time points. The results indicated that, within the nonautistic group, SA remained stable over time; conversely, in the autistic group, SA showed a decline over the same period. The main differences between the two studies lie in data collection time, population, and cultural differences. In terms of data collection time, Day et al.'s study densely sampled between June and December 2020, reflecting changes in internalized symptoms specific to COVID-19 in the relatively short term, while this study focused on the changes in SA among students returning to university campuses over a year after the normalization of the epidemic. In terms of population, Day et al.'s study mainly examined the population clinically diagnosed with ASD in comparison with those without ASD, while this study mainly reflected the general population, corresponding to the nonautistic rather than the autistic group in their study. Therefore, for those nonautistic youth, there may not be significant changes within 6 months, but changes were captured within a year, with those with higher ATs experiencing a slower decline. In terms of culture, Day et al.'s research mainly focused on the changes of young people in New York under the Western cultural background, while this study mainly focused on the changes of college students under the Eastern cultural background. In the Chinese cultural context, harmony within the group has the highest priority, and keeping consistency with other members is an important means of harmony [80]. Therefore, the difference in social communication leads to greater social pressure and higher levels of SA in individuals with high ATs. In addition, Chinese people are more sensitive to judgements from others and are more concerned about their performance in front of others [81]. Individuals exhibiting high levels of ATs tend to demonstrate elevated levels of SA, which can be attributed to their diminished capacity for cognitive empathy [39], which made it difficult to accurately infer other people's views on themselves. Therefore, in this study, it was observed that ATs hinder the decrease of SA.

### 4.3. The Longitudinal Mediating Role of Rumination Between ATs and SA

The most important result of this study, that rumination mediates longitudinally between ATs and SA, is similar to previous cross-sectional findings, which revealed the mediating effect of rumination between ATs and mood symptoms [44]. The present study specifically indicated that individuals with high levels of ATs who exhibited elevated initial levels of rumination also exhibited correspondingly higher initial levels of SA. Furthermore, individuals with high levels of ATs who demonstrated slower declines in rumination also experienced slower reductions in their levels of SA. Previous research has found that rumination is a risk factor for depression and anxiety [82, 83]. Results from several longitudinal studies have also shown that rumination levels predicted SA after periods ranging from 1 week to 1 year, for both adolescents and adults [50, 84, 85]. However, all of these studies only reflect the effect of rumination on the final outcome of SA over a certain time period, not its effect on the trajectory of SA. The present study used an LGM to analyze this problem in more depth and proposes a new interpretation of the relationship between ATs, rumination, and SA.

It should be pointed out that in East Asian culture, individuals often engage in dialectical thinking, perceiving states and objects as changeable [86]. Conversely, in Europe and America, analytical thinking is prevalent, with an expectation of a more stable world [87]. East Asians are more inclined to perceive flexibility in personal attributes and behavior compared to Westerners [88]. This leads them to view setbacks as opportunities for growth and adopt a rumination thinking to address similar challenges in the future [89]. Previous research has indicated that European Americans demonstrate lower levels of rumination compared to Asian Americans. Moreover, the relationship between rumination and adjustment—such as depressive and anxious symptoms—appears to be significantly weaker among Asian Americans than their European American counterparts [90]. The reason may be that sometimes East Asians engage in rumination for the purpose of self-improvement rather than self-doubt [89]. Further investigation is needed to assess how the longitudinal relationship between rumination and SA differs from the findings of this study in Western cultural contexts.

### 4.4. Limitations

This study has several limitations that could be addressed in future research. First, the number of follow-ups was too limited to explore the possible growth curve trends. Future studies could use latent growth curve models to explore the trajectories of curved growth of these variables based on more numerous follow-ups. Second, this study did not sample at key time points, such as the beginning of school and graduation, which is not conducive for examining the effects of key points at different stages of college on the level of SA. Lei et al. [91] found that the levels of SA in December immediately after college entry and in March of the second year were significantly higher than that within 2 weeks of the beginning of school. Future studies should further capture the relationship between the critical time points of the beginning of school and graduation and SA among college students. Third, this study only examined the changes in the college student population over the course of 1 year, and it is difficult to reveal the overall trajectories in their ATs, rumination, and SA during their university years. Future research should record the data of participants from freshmen to graduates on the above variables to address this issue. Fourth, although the relationship between ATs/rumination and SA may be influenced by Chinese culture, this study itself cannot answer whether these relationships differ between Chinese and Western cultural backgrounds, and further cross-cultural research is needed in the future. Fifth, the first time point of our research collection was in the autumn semester of 2020. In the spring semester of 2020, most universities in China adopted remote teaching, so after a semester of reduced social activities, the level of SA among college students returning to campus may be relatively high. Therefore, changes throughout the year are more easily observed, and whether this will also be seen at other time points remains to be further verified in the future. Sixth, more than two-thirds of the sample were female, although no significant differences in the variable scores based on sex were identified, and the inclusion of sex variables in LGM and SEM did not reveal any impact of sex (see Section 2 of Suporting Information S1). Future research should use a more representative large sample with a balanced sex ratio to further validate the conclusions of this study. Seventh, considering that depression is prevalent among Chinese college students [92] and that SA is often comorbid with depression [93] and bipolar disorder [94], it is important to note that other mood symptoms may introduce bias into the results, particularly when a depression dimension is included in RRS. Therefore, it should be caution when drawing conclusions. Future research should reevaluate the longitudinal relationships among ATs, rumination, and SA after controlling for the influence of these mood symptoms. Eighth, although the theoretical significance is paramount [95], the fitting indices also provide us with important references for model fitting. However, the CFA results of BAPQ suggest that its construct validity was only acceptable but not good enough. Therefore, in future research, questionnaires with better psychometric properties should be selected to measure ATs. Finally, given that modeling variability and changes in psychological constructs presents significant challenges, future research would benefit from employing multiple-indicator growth models instead of first-order (single-indicator) LGMs. This approach will facilitate a clear distinction between true variability and growth and random measurement errors [96].

## 5. Conclusion

As stable personality traits, ATs were positively associated with higher levels and slower rates of decline of rumination and SA symptoms. Furthermore, the higher levels and slower decline rates of rumination mediated the relationship between ATs and SA symptoms. Therefore, targeting rumination by psychosocial interventions may help to reduce SA symptoms and accelerate the rate of decline of SA symptoms for college students with high levels of ATs.

## Figures and Tables

**Table 1:** Descriptive statistics and bivariate correlations for variables (*n* = 397).

Variables	1	2	3	4	5	6	7	8	9
1. T1 BAPQ	1	—	—	—	—	—	—	—	—
2. T2 BAPQ	0.76*⁣*^*∗∗∗*^	1	—	—	—	—	—	—	—
3. T3 BAPQ	0.69*⁣*^*∗∗∗*^	0.80*⁣*^*∗∗∗*^	1	—	—	—	—	—	—
4. T1 RRS	0.34*⁣*^*∗∗∗*^	0.30*⁣*^*∗∗∗*^	0.25*⁣*^*∗∗∗*^	1	—	—	—	—	—
5. T2 RRS	0.33*⁣*^*∗∗∗*^	0.42*⁣*^*∗∗∗*^	0.33*⁣*^*∗∗∗*^	0.61*⁣*^*∗∗∗*^	1	—	—	—	—
6. T3 RRS	0.22*⁣*^*∗∗∗*^	0.34*⁣*^*∗∗∗*^	0.36*⁣*^*∗∗∗*^	0.57*⁣*^*∗∗∗*^	0.65*⁣*^*∗∗∗*^	1	—	—	—
7. T1 SAD-D	0.66*⁣*^*∗∗∗*^	0.57*⁣*^*∗∗∗*^	0.51*⁣*^*∗∗∗*^	0.40*⁣*^*∗∗∗*^	0.34*⁣*^*∗∗∗*^	0.26*⁣*^*∗∗∗*^	1	—	—
8. T2 SAD-D	0.49*⁣*^*∗∗∗*^	0.67*⁣*^*∗∗∗*^	0.56*⁣*^*∗∗∗*^	0.37*⁣*^*∗∗∗*^	0.48*⁣*^*∗∗∗*^	0.41*⁣*^*∗∗∗*^	0.64*⁣*^*∗∗∗*^	1	—
9. T3 SAD-D	0.44*⁣*^*∗∗∗*^	0.54*⁣*^*∗∗∗*^	0.64*⁣*^*∗∗∗*^	0.30*⁣*^*∗∗∗*^	0.32*⁣*^*∗∗∗*^	0.44*⁣*^*∗∗∗*^	0.58*⁣*^*∗∗∗*^	0.61*⁣*^*∗∗∗*^	1
*M*	82.44	83.50	82.96	47.62	47.07	46.51	12.44	12.02	11.64
*SD*	15.88	16.40	16.16	11.23	12.70	12.27	7.30	7.68	7.07

Abbreviations: BAPQ, Broad Autism Phenotype Questionnaire; RRS, Ruminative Response Scale; SAD-D, Social Anxiety Disorder Dimension.

*⁣* ^*∗∗∗*^ *p* < 0.001.

**Table 2:** Measurement invariance across different time points.

Variables	Model	CFI	TLI	RMSEA	ΔCFI	ΔTLI	ΔRMSEA
BAPQ	Configural invariance	0.878	0.866	0.039	—	—	—
	Metric invariance	0.877	0.867	0.038	−0.001	0.001	−0.001
	Scalar invariance	0.873	0.865	0.039	−0.004	−0.002	0.001
	Strict invariance	0.871	0.865	0.039	−0.002	0	0

RRS	Configural invariance	0.925	0.916	0.035	—	—	—
	Metric invariance	0.924	0.917	0.034	−0.001	0.001	−0.001
	Scalar invariance	0.922	0.917	0.034	−0.002	0	0
	Strict invariance	0.920	0.917	0.034	−0.002	0	0

SAD-D	Configural invariance	0.974	0.968	0.032	—	—	—
	Metric invariance	0.975	0.970	0.031	0.001	0.002	−0.001
	Scalar invariance	0.970	0.966	0.033	−0.005	−0.004	0.002
	Strict invariance	0.970	0.967	0.032	0	0.001	−0.001

Abbreviations: BAPQ, Broad Autism Phenotype Questionnaire; CFI, comparative fit index; RMSEA, root mean square error of approximation; RRS, Ruminative Response Scale; SAD-D, Social Anxiety Disorder Dimension; TLI, Tucker–Lewis index.

**Table 3:** Unconditional latent growth models of variables.

Variables	*χ* ^2^	*df*	RMSEA	CFI	TLI	Intercept^a^	Slope^a^	Intercept-slope covariance^b^
BAPQ	8.296	2	0.089	0.977	0.965	82.44*⁣*^*∗∗∗*^	0.24	−0.36*⁣*^*∗∗∗*^
RRS	8.950	2	0.094	0.978	0.968	47.62*⁣*^*∗∗∗*^	−0.56*⁣*^*∗*^	−0.40*⁣*^*∗∗∗*^
SAD-D	8.167	2	0.088	0.975	0.963	12.44*⁣*^*∗∗∗*^	−0.40*⁣*^*∗*^	−0.55*⁣*^*∗∗∗*^

Abbreviations: BAPQ, Broad Autism Phenotype Questionnaire; CFI, comparative fit index; RMSEA, root mean square error of approximation; RRS, Ruminative Response Scale; SAD-D, Social Anxiety Disorder Dimension; TLI, Tucker–Lewis index.

^a^Nonstandardized results.

^b^Standardized results.

*⁣* ^*∗*^ *p* < 0.05.

*⁣* ^*∗∗∗*^ *p* < 0.001.

## Data Availability

The data that support the findings of this study are available from the corresponding author upon reasonable request.

## Supporting Information

Supporting Information Supporting Information S1 include two sections. Section 1 presents the results after excluding seven participants who were in the gap year before graduate school. Section 2 presents the results related to the sex differences.
