# Supplementary material for: Autistic Traits and Social Anxiety in Chinese College Students: The Longitudinal Mediating Role of Rumination
Source: Depress Anxiety. 2025 Oct 1;2025:6103362. doi: 10.1155/da/6103362 (PMC12507499; doi:10.1155/da/6103362)
Supplement: Supporting Information — S1 include two sections. Section 1 presents the results after excluding seven participants who were in the gap year before graduate school. Section 2 presents the results related to the sex differences. [file 6103362.f1.docx]

**Supplementary materials**

**Autistic traits and social anxiety in Chinese college students: The longitudinal mediating role of rumination**

**Section 1. The results without 7 participants in the gap year**

***1.1 Trajectories of ATs, rumination, and SA***

Results from the latent growth models for the trajectories of ATs, rumination, and SA are presented in **Table S1**. These results showed that the fit indices for the three models were good, with the exception of the RMSEA. The slope for the ATs was not significant, indicating that no significant linear change in ATs occurred among college students between the three measurements. The slopes of rumination and SA were negative, indicating a decrease in rumination and SA during the study.

**Table S1** Unconditional latent growth models of ATs, rumination and SA

|  | χ^2^ | df | RMSEA | CFI | TLI | Intercept ^a^ | Slope ^a^ | Intercept-slope covariance ^b^ |
| --- | --- | --- | --- | --- | --- | --- | --- | --- |
| BAPQ | 8.796 | 2 | .093 | .974 | .961 | 82.57^***^ | 0.25 | -.36^***^ |
| RRS | 8.638 | 2 | .092 | .979 | .969 | 47.72^***^ | -0.61^*^ | -.41^***^ |
| SAD-D | 7.950 | 2 | .087 | .976 | .963 | 12.54^***^ | -0.41^*^ | -.54^***^ |

*Note*. BAPQ = Broad Autism Phenotype Questionnaire, RRS = Ruminative Response Scale, SAD-D = Social Anxiety Disorder Dimension; RMSEA = root mean square error of approximation, CFI = comparative fit index; TLI = Tucker-Lewis index; ^***^*p* < .001; ^a^ Non-standardized results, ^b^ Standardized results.

***1.2 The direct effects of ATs on the trajectory of SA***

Due to the nonsignificant slope of ATs in the unconditional latent growth model, we subsequently used the conditional latent growth model rather than parallel latent growth model to model the effect of the baseline ATs levels on the trajectory SA.

The conditional latent growth model with ATs at T1 as the independent variable, and the trajectory of SA as the dependent variable was found to fit well *(χ^2^*/*df* =3.35, RMSEA = .078, CFI = .985, TLI = .970). ATs positively predicted the intercept of SA (*β* = .66, *t* = 23.46, *p* < .001), and negatively predicted the slope of SA (*β* = -.28, *t* = -6.05, *p* < .001).

- 1. ***The longitudinal mediating role of rumination between ATs and SA***

The results of the structural equation model showed good fit indices (*χ^2^*/*df* = 3.82, CFI = .973, TLI = .943, RMSEA = .085). ATs positively predicted the intercept (*β* = .59, *t* = 11.35, *p* < .001), and negatively predicted the slope (*β* = -.25, *t* = -1.78, *p* = .075) of SA. Meanwhile, ATs positively predicted the intercept (*β* = .43, *t* = 7.18, *p* < .001), and negatively predicted the slope (*β* = -.18, *t* = -2.26, *p* = .02) of rumination. The intercept of rumination positively predicted the intercept (*β* = .32, *t* = 4.94, *p* < .001), but did not significantly predict the slope (*β* = -.06, *t* = -0.76, *p* = .45) of SA. Finally, the slope of rumination positively predicted the slope (*β* = .90, *t* = 3.49, *p* < .001), but not the intercept (*β* = -.06, *t* = -0.76, *p* = .45) of SA.

Furthermore, the Bootstrap results showed that mediating effect of the indirect pathways of ATs → rumination intercept → SA intercept was .14, 95% CI [.08 .23], and that mediating effect of the indirect pathways of ATs → rumination slope → SA slope was -.16, 95% CI [-.54 -.003]. Because the 95% CI did not include 0, the two indirect pathways were both significant.

**Section 2. The results related to the sex differences**

***2.1 The sex differences on the variable scores***

As shown in the **Table S2**, the independent sample *t*-tests results showed that there were no significant differences between male and female students in all variables (*p*s > .05).

**Table S2** The sex differences on the variable scores

| Variables | Male (*n* = 125) | Female (*n* = 272) | *t* | *p* |
| --- | --- | --- | --- | --- |
| T1 BAPQ | 82.75 ± 15.24 | 82.29 ± 16.19 | 0.27 | .79 |
| T2 BAPQ | 82.86 ± 17.10 | 83.79 ± 16.10 | -0.52 | .60 |
| T3 BAPQ | 81.34 ± 16.88 | 83.70 ± 15.79 | -1.36 | .18 |
| T1 RRS | 47.17 ± 11.27 | 47.83 ± 11.23 | -0.55 | .59 |
| T2 RRS | 46.82 ± 13.61 | 47.18 ± 12.29 | -0.27 | .79 |
| T3 RRS | 45.36 ± 13.23 | 47.04 ± 11.80 | -1.27 | .21 |
| T1 SAD-D | 12.70 ± 7.04 | 12.32 ± 7.43 | 0.48 | .63 |
| T2 SAD-D | 12.02 ± 7.86 | 12.03 ± 7.61 | -0.01 | .99 |
| T3 SAD-D | 11.64 ± 6.97 | 11.64 ± 7.13 | <0.001 | 1.00 |

*Note*. BAPQ = Broad Autism Phenotype Questionnaire, RRS = Ruminative Response Scale, SAD-D = Social Anxiety Disorder Dimension.

***2.2 The direct effects of ATs on the trajectory of SA***

The conditional LGM with ATs at T1 and sex as the independent variable, and the trajectory of SA as the dependent variable was found to fit well *(χ^2^*/*df* = 2.56, RMSEA = .063, CFI = .988, TLI = .974). ATs positively predicted the intercept of SA (*β* = .66, *t* = 23.83, *p* < .001), and negatively predicted the slope of SA (*β* = -.29, *t* = -6.16, *p* < .001). However, the effect of sex on the intercept (*β* = -.02, *t* = -0.44, *p* = .66) and slope (*β* = .03, *t* = 0.54, *p* = .59) of SA were insignificant.

- 1. ***The longitudinal mediating role of rumination between ATs and SA***

The results of the SEM showed good fit indices (*χ^2^*/*df* = 3.32, CFI = .975, TLI = .943, RMSEA = .076). ATs positively predicted the intercept (*β* = .59, *t* = 11.56, *p* < .001), and negatively predicted the slope (*β* = -.27, *t* = -2.01, *p* = .045) of SA. Meanwhile, ATs positively predicted the intercept (*β* = .44, *t* = 7.29, *p* < .001), and negatively predicted the slope (*β* = -.17, *t* = -2.11, *p* = .04) of rumination. The intercept of rumination positively predicted the intercept (*β* = .32, *t* = 4.98, *p* < .001), but did not significantly predict the slope (*β* = -.08, *t* = -0.65, *p* = .52) of SA. Finally, the slope of rumination positively predicted the slope (*β* = .86, *t* = 3.59, *p* < .001), but not the intercept (*β* = -.07, *t* = -0.91, *p* = .36) of SA. However, the effect of sex on the intercept (*β* = -.02, *t* = -0.48, *p* = .63) and slope (*β* = -.02, *t* = -0.19, *p* = .85) of SA, as well as the intercept (*β* = .03, *t* = 0.50, *p* = .62) and slope (*β* = .07, *t* = 0.89, *p* = .37) of rumination were insignificant.

Furthermore, the Bootstrap results showed that the mediating effect of the indirect pathways of ATs → rumination intercept → SA intercept was .14, 95% CI [.08 .22], and that mediating effect of the indirect pathways of ATs → rumination slope → SA slope was -.14, 95% CI [-.47 -.001]. Because the 95% CI did not include 0, the two indirect pathways were both significant.
